# An automatic method for identifying surface proteins in bacteria: SLEP

**DOI:** 10.1186/1471-2105-11-39

**Published:** 2010-01-20

**Authors:** Emanuela Giombini, Massimiliano Orsini, Danilo Carrabino, Anna Tramontano

**Affiliations:** 1 Department of Biochemical Sciences "A. Rossi Fanelli", Sapienza University. 00185 Rome, Italy; 2 Center for Advanced Studies, Research and Development in Sardinia (CRS4), 09010 Pula, Italy; 3 CASPUR, Consorzio Interuniversitario per le Applicazioni di Supercalcolo per Universita' e Ricerca, 00185 Rome, Italy; 4 Istituto Pasteur Fondazione Cenci - Bolognetti, Sapienza University. 00185 Rome, Italy

## Abstract

**Background:**

Bacterial infections represent a global health challenge. The identification of novel antibacterial targets for both therapy and vaccination is needed on a constant basis because resistance continues to spread worldwide at an alarming rate. Even infections that were once easy to treat are becoming difficult or, in some cases, impossible to cure. Ideal targets for both therapy and vaccination are bacterial proteins exposed on the surface of the organism, which are often involved in host-pathogen interaction. Their identification can greatly benefit from technologies such as bioinformatics, proteomics and DNA microarrays.

**Results:**

Here we describe a pipeline named SLEP (Surface Localization Extracellular Proteins), based on an automated optimal combination and sequence of usage of reliable available tools for the computational identification of the surfome, i.e. of the subset of proteins exposed on the surface of a bacterial cell.

**Conclusions:**

The tool not only simplifies the usage of these methods, but it also improves the results by selecting the specifying order and combination of the instruments. The tool is freely available at http://www.caspur.it/slep.

## Background

Bacterial diseases are among the major causes of mortality and morbidity in humans. Antibiotics are the first line of defence against bacteria, however more and more bacteria are antibiotic resistant and the phenomenon is spreading at an alarming rate [1,2]. Many diseases are becoming increasingly difficult to fight. There are several examples of microbial infections that are becoming resistant to all existing therapies and for which a vaccination strategy is deemed to be appropriate, such as gonorrhea, tuberculosis, pneumonia, septicaemia and childhood ear infections [3-7].

Among the proteins encoded by bacteria, secreted and surface proteins are particularly important in bacterial pathogenesis. The former can be involved in host cell toxicity and lead to more or less subtle alterations of the host cell for the benefit of the pathogen. Bacterial surface proteins play a fundamental role in the interaction with the cell environment [8-12]. They can be involved in adhesion and invasion of the host cells as well as in defending against host responses. Because of this, surface proteins are potential drug targets [13]. Moreover, surface proteins are likely to interact with the host immune system and are ideal candidates for vaccine development [14-16].

Surface proteins include integral or transmembrane proteins that span the membrane and have a hydrophilic cytosolic domain, which interacts with internal molecules, a hydrophobic membrane-spanning domain that anchors it within the cell membrane, and a hydrophilic extracellular domain that interacts with external molecules. Lipid anchored proteins are instead covalently-bound to one or more lipid molecules. Other membrane proteins are peripheral, i.e. they are attached to integral membrane proteins, or associated with regions of the lipid bilayer.

Gram-positive bacteria possess a thick cell wall containing many layers of peptidoglycan and teichoic acids. In contrast, Gram-negative bacteria have a relatively thin cell wall consisting of a few layers of peptidoglycan surrounded by a second lipid membrane containing lipopolysaccharides and lipoproteins. This is reflected in their membrane protein composition. Cell wall proteins are found in Gram+ bacteria while β-barrel membrane proteins are only found in the outer membranes of Gram- organisms, in mitochondria and chloroplast [17].

Despite the biological relevance of bacterial surface proteins, their characterization is still incomplete. There are two main routes to identify surface proteins. In one approach, membrane and cell wall fractions are separated from the cytoplasmic fraction and then proteins are identified by two-dimensional (2D)-electrophoresis or 2D-chromatography coupled to mass spectrometry [see for example [18-23]]. The other possibility is to take advantage of bioinformatics and attempt their prediction on the basis of one of the many specifically developed algorithms.

There is a plethora of available tools for predicting the membrane localization and topology of a protein and the presence of specific localization signals in its sequence, but not every method is equally accurate and, especially, an end user is not always well informed about novel developments in the field. The order in which these tools are used might also make a difference, as we will show here. Furthermore, each of them tends to use different input formats and not always self explanatory output formats.

The aim of the work described here is to bring these tools in a coordinated and easy-to use form to the bench scientists who, on one side, should not need to be familiar with the ins and outs of each and every tool, but, on the other, should be given sufficient information to assess the reliability of the methods.

## Implementation

SLEP and all the related tools have been implemented locally on a linux SLES 10 server.

The programs included in the SLEP automatic procedure are Glimmer [24-26], TMHMM [27,28], prodiv-HMM [29,30], pSORTb [31,32] and LipoP [30] all ran with default parameters.

If the user inputs a genome, putative genes need to be identified and translated into their amino acid sequence. This is achieved using Glimmer, a gene finding program based on Interpolated Markov Models (IMMs) [24-26]. The accuracy of gene identification by Glimmer depends upon the length and the GC-content the genome and is reported at http://www.cbcb.umd.edu/software/glimmer/.

The translated gene products, or the input proteins (if the user selected to start with a known proteome) are analysed for the presence of transmembrane regions using TMHMM [27,28] and prodiv-HMM [29], two independent Hidden Markov Model-based prediction methods. It is a known problem in the field that signal peptides might often be mispredicted as transmembrane helices and vice versa. To alleviate this problem, we only assign the "membrane protein" tag to proteins for which more than three transmembrane helix are predicted by at least one method. As described later, proteins for which no signal peptide is identified are re-submitted to the transmembrane prediction tools.

Proteins not assigned to the "membrane" bin are analysed using LipoP [30], a tool for identifying signal peptides of both type I and II in a protein sequence. Because all clearly detectable membrane proteins have been already filtered out in the previous step, the number of false positives, i.e. the number of times LipoP predicts as a signal peptide what is in reality a transmembrane helix, is reduced. Table 1 shows the comparison between the accuracy obtained using LipoP on the complete dataset and that achieved by running it only on the filtered set of proteins, i.e. on proteins not including predicted transmembrane proteins with three or more helices, according to the SLEP protocol. The decrease in the number of false positives, although rather small, justifies our choice in using the tool only after filtering out the predicted multiple membrane spanning proteins.

**Table 1:** Comparison between SLEP and LipoP

	TP	FP	FN
***Gram*+**			

**LipoP**	227	47	5
**LipoP/SLEP**	227	19	5

***Gram*-**			

**LipoP**	266	45	5
**LipoP/SLEP**	266	43	5

Comparison of the accuracy of LipoP [30] ran on the whole set of proteins and on a reduced set after removing predicted transmembrane proteins with three or more helices according to the SLEP protocol.

The next step consists in running pSORTb [31,32] on the remaining set of proteins. pSORTb is used for recognizing cell wall proteins (in Gram+ bacteria) and outer membrane proteins (in Gram- bacteria) as well as exported proteins. The remaining proteins are reanalysed by TMHMM and prodiv-HMM in order to identify proteins with a single membrane spanning helix. As mentioned before, we remove clearly detectable membrane proteins before attempting the prediction of the presence of signal peptides. Only if no signal peptide has been identified in the sequence, we look for single membrane spanning helix.

The statistical parameters used for evaluating the accuracy of the predictions are:

Where TP, TN, FP and FN are the number of True Positive, True Negative, False Positive and False Negative results, respectively.

## Results and discussion

**Figure 1:**
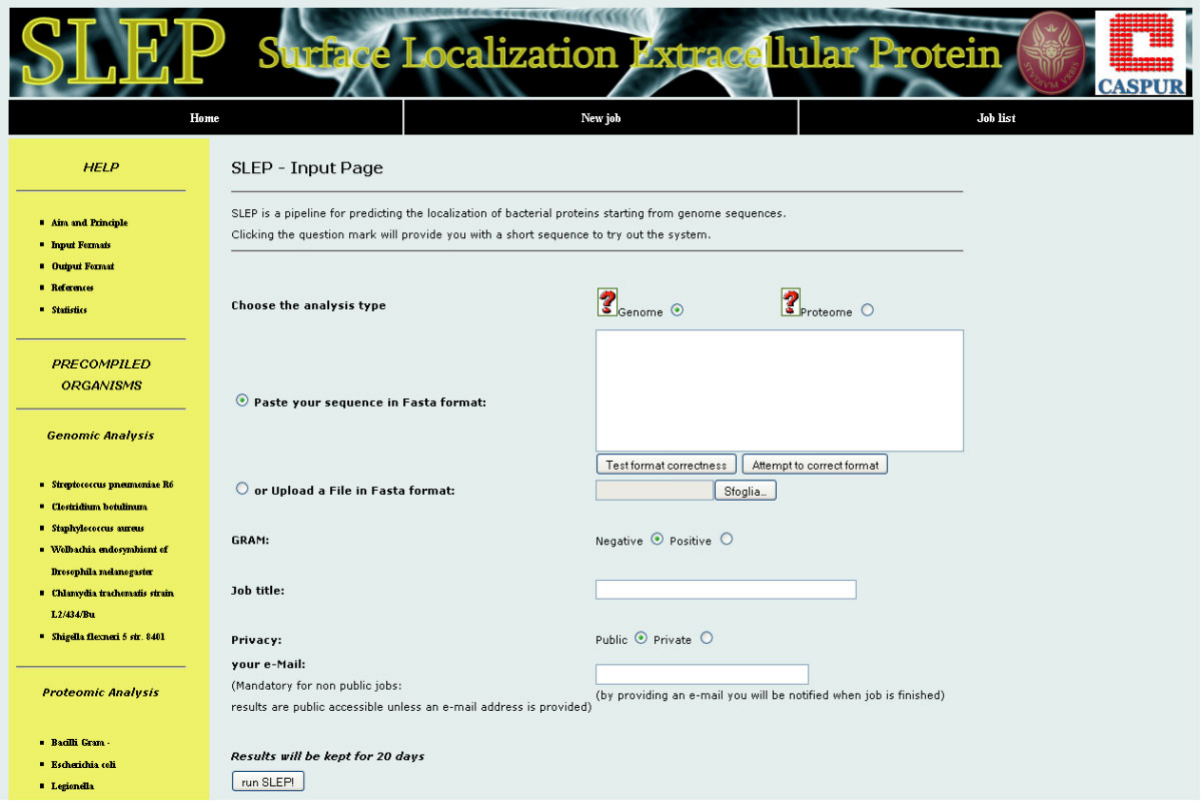
**SLEP home page**. Graphics interface of the initial submission page of SLEP.

SLEP is based on an automated optimal combination and succession of usage of some of the most reliable available tools. The user needs to input either the genomic sequence of the bacterial organisms under study or its proteome together with the information of whether the bacterium is Gram+ or Gram-. The main purpose of SLEP is to provide users with an easy-to-use tool for the prediction of protein localization with the highest possible accuracy achievable today. The user interface of the tool is illustrated in Figure 1.

**Figure 2:**
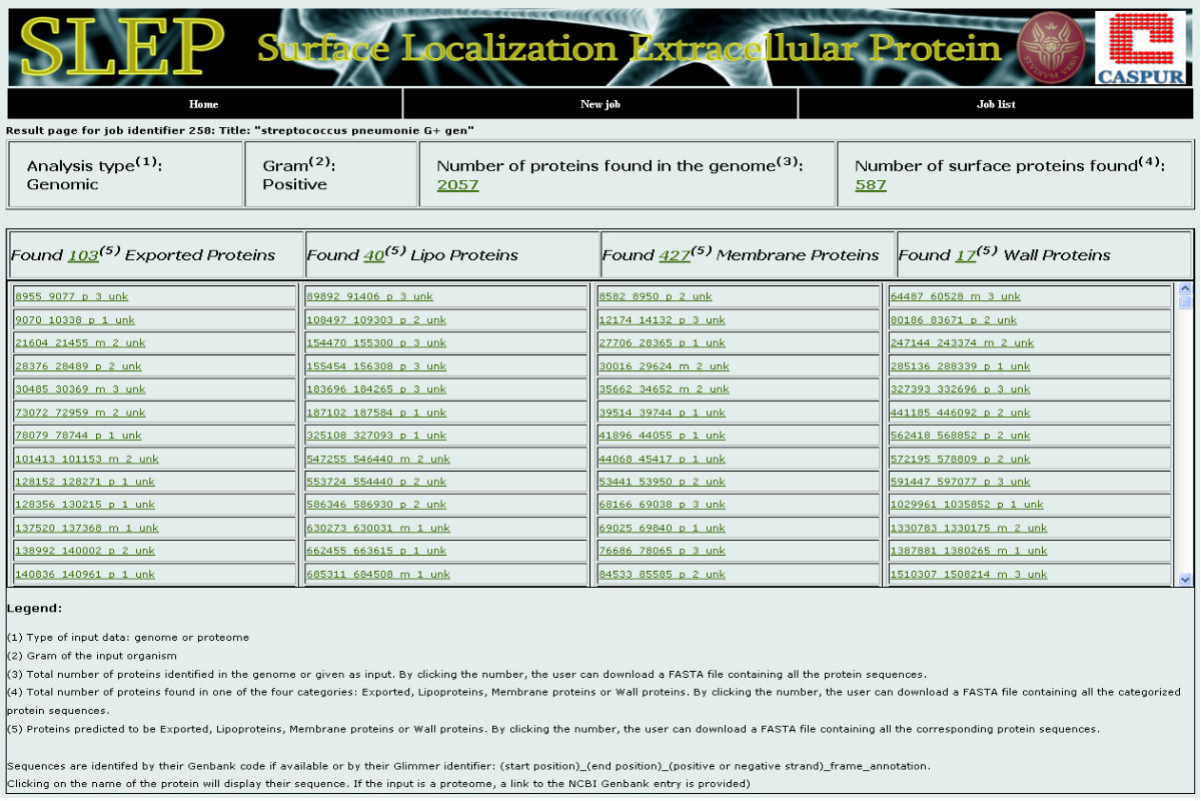
**SLEP output page**. An example of a SLEP output page.

The output of the system is an organized list of proteins (or putative proteins if the input is a genome) classified according to their predicted localization. In particular it will separately list lipoproteins, membrane, exported and secreted proteins, cell wall proteins or outer membrane proteins in Gram+ or Gram- bacteria, respectively (Figure 2).

We tested the accuracy of the procedure using the manually curated database SwissProt. This dataset, named SP, contained 18,510 protein sequences of known localization (as reported in the SUBCELLULAR LOCATION field), roughly equally populated by protein from Gram- and Gram+ bacteria (9,946 and 9,564, respectively). For Gram+ bacteria we used Enterococcus (EN, 228 proteins), Listeria (LI, 749 proteins), Staphylococcus (SP, 3981 proteins), Streptococcus (ST, 179 proteins) and a pool of Bacilli Gram+ organisms (B+, 4427 proteins). For Gram-, the datasets contained data from E. coli (EC, 3891 proteins), Legionella (LE, 421 proteins), Pseudomonas (PS, 3369 proteins), Salmonella (SA, 2019 proteins) and a pool of Bacilli Gram- organisms (B-, 246 proteins).

The overall accuracy of the predictions that can be achieved in a single click using SLEP is illustrated in Table 2.

**Table 2:** SLEP overall accuracy

	Gram+ Total number of proteins: 9,564			
	**MEMBRANE**	**LIPOPROTEINS**	**EXPORTED**	**CELL WALL**

**TP**	2057	227	279	118

**FP**	76	19	221	29

**TN**	7335	9313	8960	9396

**FN**	96	5	104	21

**SE**	95.5	97.8	72.8	84.9

**SP**	99.0	99.8	97.6	99.7

**PPV**	96.4	92.3	55.8	80.3

**NPV**	98.7	99.9	98.9	99.8

**Accuracy**	98.2	99.7	96.6	99.5

**CCM**	94.8	94.9	62.0	82.3

	**Gram-. Total number of proteins: 9,946**			

	**MEMBRANE**	**LIPOPROTEINS**	**EXPORTED**	**OUTER MEMBRANE**

**TP**	2743	266	711	179

**FP**	57	40	224	46

**TN**	6971	9635	8831	9696

**FN**	175	5	180	25

**SE**	94.0	98.2	80.2	87.7

**SP**	99.2	99.6	97.5	99.5

**PPV**	98.0	86.9	75.9	79.6

**NPV**	97.6	99.9	98.1	99.7

**Accuracy**	97.7	99.5	96.0	99.3

**CCM**	94.3	92.1	75.8	83.2

Overall accuracy of SLEP for Gram+ and Gram- bacteria. See Material and Methods for the definition of the parameters.

In Table 3 we compare our results with the use of PSORTb alone. For completeness, we report in the same Table the accuracy of other available methods for the relevant datasets [33-38]. Notice that the tools included in SLEP have been selected for their accuracy, but also for their availability as stand-alone programs since they are all implemented locally to speed up the procedure.

**Table 3:** Comparison between SLEP and other available tools

	Gram+				Gram-				
	
	SLEP	PSORTb	PHOBIUS	PRED-LIPO	SLEP	PSORTb	PHOBIUS	PRED-LIPO	ProfTMB
	
Membrane	98.6	91.8			97.8	92.7			
**Lipoproteins**	99.8	98.5		99.4	99.6	98.4		98.9	

**Exported**	96.6	97.8	93.8		95.7	94.9	90.5		

**Cell wall/Outer membrane**	99.7	99.6			99.5	98.9			97.8

Comparison of the accuracy of SLEP, PSORTb [31,32], PHOBIUS [33-35], PRED-LIPO [36] and Prof-TMB [37,38] on our dataset. For Prof-TMB, we only considered as positive proteins for which at least 14 transmembrane segments were predicted as this choice substantially increases the accuracy of the method.

## Conclusions

Bioinformatics tools are extremely useful for the bench scientists and most of them are mature enough to be considered part of a toolbox that should be readily and easily accessible to all.

The appropriate usage of the tools is however essential. This is far from being trivial: one of the most cogent problems in bioinformatics is that way too often obsolete tools remain available and are used by experimentalists who are unaware of more recent developments. Users are confronted with too many available tools, not all properly benchmarked and updated and this can result in a waste of time and effort. The problem is even more relevant when the methods need to be used as start points of a set of experiments where an incorrect selection/usage of the methods can seriously affect the end results.

The initial selection of the set of transcripts/proteins from a pathogen to be used as targets for the development of vaccines and/or inhibitor screening is one such case and yet no comprehensive easy-to-use system was available so far. Perhaps the most complete resource available is Augur [39] which includes a precompiled list of protein localizations and other useful features, but does not allow users to supply their own genome/proteome or set of proteins as is the case in SLEP and is limited to Gram negative bacteria.

We have described here an automatic procedure designed to achieve an accurate prediction of bacterial protein localization via an appropriate sequence of usage of the available methods that is, at the same time, extremely easy to use.

SLEP uses a combination of state of the art methods that have been shown to be the most accurate available [29,30,32]. The specific order of usage of these programs has been designed to reduce the chance of misclassification by each of the tools.

The system relieves the bench scientists from the burden of selecting the most accurate programs for the task at hand. SLEP will be continuously updated to reflect novel developments and plans to be the one-stop shop for the analysis of bacterial protein localization that is perhaps the most important aspect of therapeutic target selection.

## Availability and requirements

**• Project name**: SLEP

**• Project home page**: http://www.caspur.it/slep

**• Operating system(s)**: Platform independent

**• Programming language**: Perl and Python

**• Any restrictions to use by non-academics**: None

## Authors' contributions

EG implemented the system. MO wrote the scripts for running glimmer. DC designed and developed the SLEP website. AT was involved in coordinating the work and in drafting the manuscript. All authors read and approved the final manuscript
